# Supplementary figures and images for: Ligand-induced IFNGR1 down-regulation calibrates myeloid cell IFNγ responsiveness
Source: Life Sci Alliance. 2019 Oct 4;2(5):e201900447. doi: 10.26508/lsa.201900447 (PMC6778285; doi:10.26508/lsa.201900447)

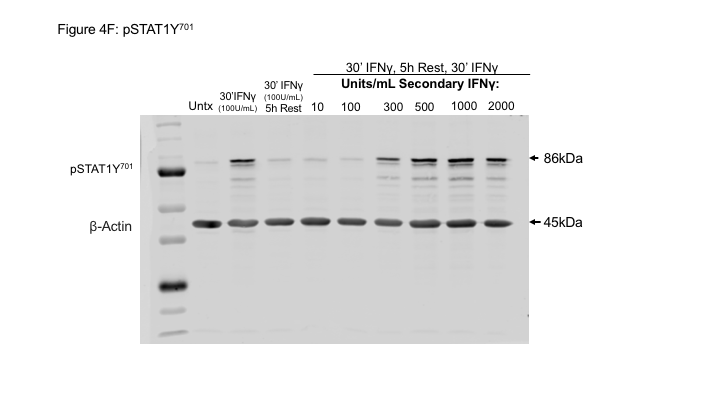

Supplement: Supplementary file 2 [file LSA-2019-00447_SdataF4b.tif]

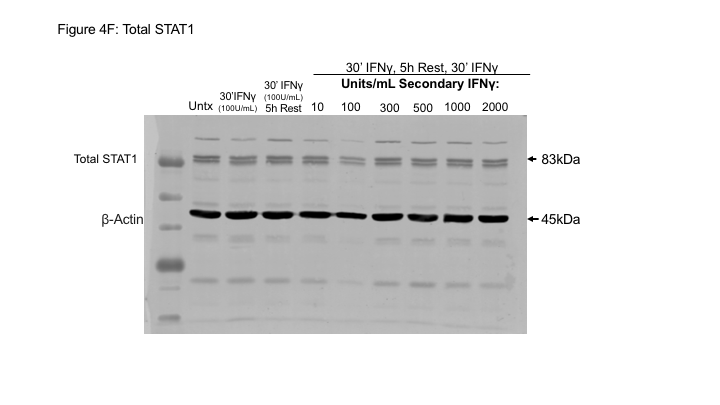

Supplement: Supplementary file 3 [file LSA-2019-00447_SdataF4c.tif]

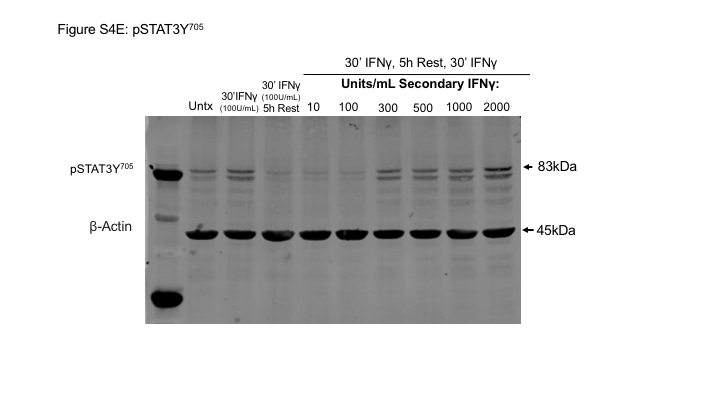

Supplement: Supplementary file 5 [file LSA-2019-00447_SdataFS4b.tif]

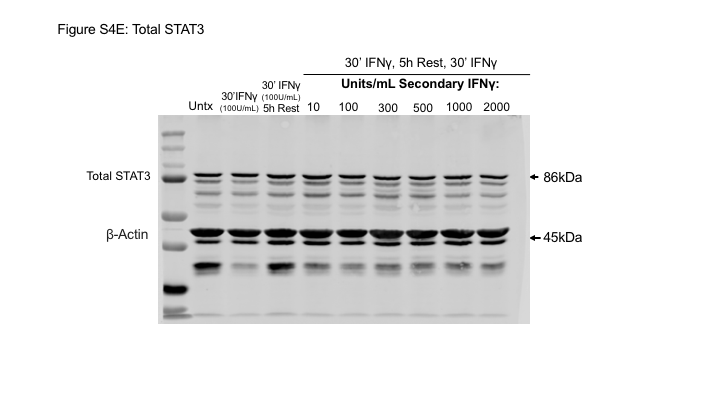

Supplement: Supplementary file 6 [file LSA-2019-00447_SdataFS4c.tif]
